# Genotypes diversity of *env* gene of *Bovine leukemia virus* in Western Siberia

**DOI:** 10.1186/s12863-020-00874-y

**Published:** 2020-10-22

**Authors:** Natalia Blazhko, Sultan Vyshegurov, Alexander Donchenko, Kirill Shatokhin, Valeria Ryabinina, Kirill Plotnikov, Alevtina Khodakova, Sergey Pashkovskiy

**Affiliations:** 1 Laboratory of Enzyme Analysis and DNA Technologies, Novosibirsk State Agricultural University, Novosibirsk, Russia; 2 Laboratory of Leukemia, Institute of experimental veterinary medicine, Siberia and the Far East, Siberian Federal Research Centre for Agrobiotechnology of the Russian Academy of Sciences, Krasnoobsk, Novosibirsk region Russia

**Keywords:** Bovine leukemia virus, Cattle, Env gene, Genotypes, Agriculture

## Abstract

**Background:**

This study describes the biodiversity and properties of *Bovine leukemia virus* in Western Siberia. This paper explores the effect of different genotypes of the *env* gene of the cattle leukemia virus on hematological parameters of infected animals. The researchers focused on exploring the polymorphism of the *env* gene and, in doing so, discovered the new genotypes *I*_*a*_ and *I*_*b*_, which differ from genotype *I*. Several hypotheses on the origin of the different genotypes in Siberia are discussed.

**Results:**

We obtained varying length of the restriction fragments for genotypes *I*_._ Additionally using restrictase *Hae III* were received fragments was named genotype *I*_*a*_, and genotype *I*_*b*_. There are 2.57 ± 0.55% (20 out of 779) samples of genotype *I*_*b*_ which does not differ significantly from 1% (χ^2^ = 2.46). Other genotypes were observed in the cattle of Siberia as wild type genotypes (their frequency varied from 17.84 to 32.73%). The maximum viral load was observed in animals with the II and IV viral genotypes (1000–1400 viral particles per 1000 healthy cells), and the minimum viral load was observed animals with genotype *I*_*b*_ (from 700 to 900 viral particles per 1000 healthy cells).

**Conclusions:**

The probability of the direct introduction of genotype *II* from South America to Siberia is extremely small and it is more likely that the strain originated independently in an autonomous population with its distribution also occurring independently. A new variety of genotype *I* (*I*_*b*_) was found, which can be both a neoplasm and a relict strain.

## Background

Viruses are the most variable form of life, and *BLV* is not an exception. The origin of new strains is a continuous process [1–4]. Monitoring the origin of new virus mutations is of great importance for veterinary and animal husbandry, as every new strain may have unique features of interaction with the host organism. Accordingly, every new strain of the virus can be cause of completely another symptoms of the disease [5]. That’s why, the monitoring of the spread and emergence of virus strains is relevant in every separated geographical region.

One of the most common and dangerous diseases of cattle is leukemia caused by *Bovine leukemia virus* (*BLV*), which belongs to the family *Retroviridae* [6, 7]. One of its features is a high proportion of latent virus carriers (70–90%) and low detestability of clinical symptoms by hematological methods, which complicates measures to identify sick animals and prevent the further spread of the disease [8–10]. *BLV* it is a virus transmitted by direct contact between animals. The transmitted *BLV* from cattle to humans is possible but clinical symptoms in *Homo sapiens* have been registered in singular cases [11].

The probable reason for this is the tendency of *Retroviridae* representatives to embed into the carrier genome [4, 12]. Therefore, it is important for viruses to have such a protein composition of the capsid, so as not to be interpreted by the immune system of the carrier as a danger. In *BLV*, capsid proteins are encoded by *env* gene. The *env* gene is located between the *pol* gene and the 3’LRT-area; it is 1547 bp in length (from nucleotides 4826 to 6373, numbering from the *BLV env* sequence of the isolate FLK-*BLV*, GenBank accession number EF600696). 4615–6160 bp). This gene encodes the glycoprotein viral membrane of *gp*51 and the transmembrane protein *gp*30. The proteins -*gp*51(SU) and *gp*30 (TM) are glycolized [13–16]. Protein *gp*30 contributes to the contact between viral particles and В- and Т- cells and its properties affect the ability of the sheep immune system to recognize the viral particles [17, 18]. This protein can define the ability of *BLV* to affect the immune system cells of a carrier [19]. The *gp*51 protein reacts with monoclonal cattle antibodies and determines the degree of *BLV* virulence [20]. Therefore, qualitative changes in the *gp*30 and *gp*51 proteins that are caused by gene mutations may become the factors that affect the intensity of the immune response. So for specialists involved in coevolution of *BLV* and cattle, it is of great interest to study the relationship between individual mutations and genotypes of the virus with the phenotypic manifestation of leukemia.

The *BLV* genome includes four structural genes (*env, pro*, *gag,* and *pol*), of which *env* is the most suitable for detecting the pathogen by PCR analysis [21–23]. There are 10 genotypes of the *env* gene in the world [24, 25] and each creates slight differences in the biological properties of the virus. This research aims to definethe virulent properties of the *BLV* genotypes that have been discovered in Western Siberia as well as the newly discovered forms of the genotypes on the *env* gene.

## Results

**Fig. 1:**
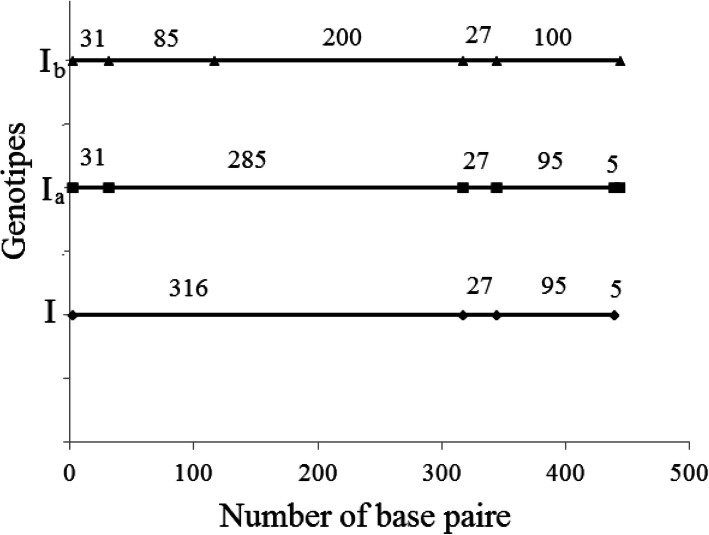
Schemeof *HaeIII* restriction with formation of genotypes

PCR-RFLP analysis of the *env* gene detected 5 genotypes that differed on the lengths of fragment restriction. This restriction is caused by restrictases *HaeIII* and *BstYI* (Table 1). The effect of the restriction endonuclease *HaeIII* differed from the supposed effect. The fragment restriction lengths of 316, 27, 95, and 5 bp were expected to show up on the electrophorogram as they are considered to be the standard products of genotype *I* restriction [26]. However, additional fragments of restriction were found as genotypes *I*_*a*_ and *I*_*b*_ (Table 1, Fig. 1).

**Table 1:** Scheme of genotypes formed by restrictases *HaeIII* and *BstYI*

Genotype	Restrictase	
	*Hae III*	*BstYI*
I	316–27–95-5	–
I_a_	31–285–27-95-5	–
I_b_	31–85–200-27-100	–
II	–	529–322-143
IV	–	672–322

The table highlights the fragment restrictions with length (b.p)

Restriction fragments (length 27 bp) are considered to be a common trait for all three genotypes of the family *I*. The common factors for genotypes *I* and *I*_*a*_ are fragments of 95 and 5 bp (Fig. 1). Genotype *I*_*b*_ differs from related genetic elements as it combines these fragments into a single one (100 bp length). The common restriction fragments of genotypes *I*_*a*_ and *I* reveal a short sequence (31 bp), whereas the long fragment that is typical for genotype *I*_*a*_ (285 bp), is represented by two separate factions (85 and 200 bp) (Fig. 1).

**Fig. 2:**
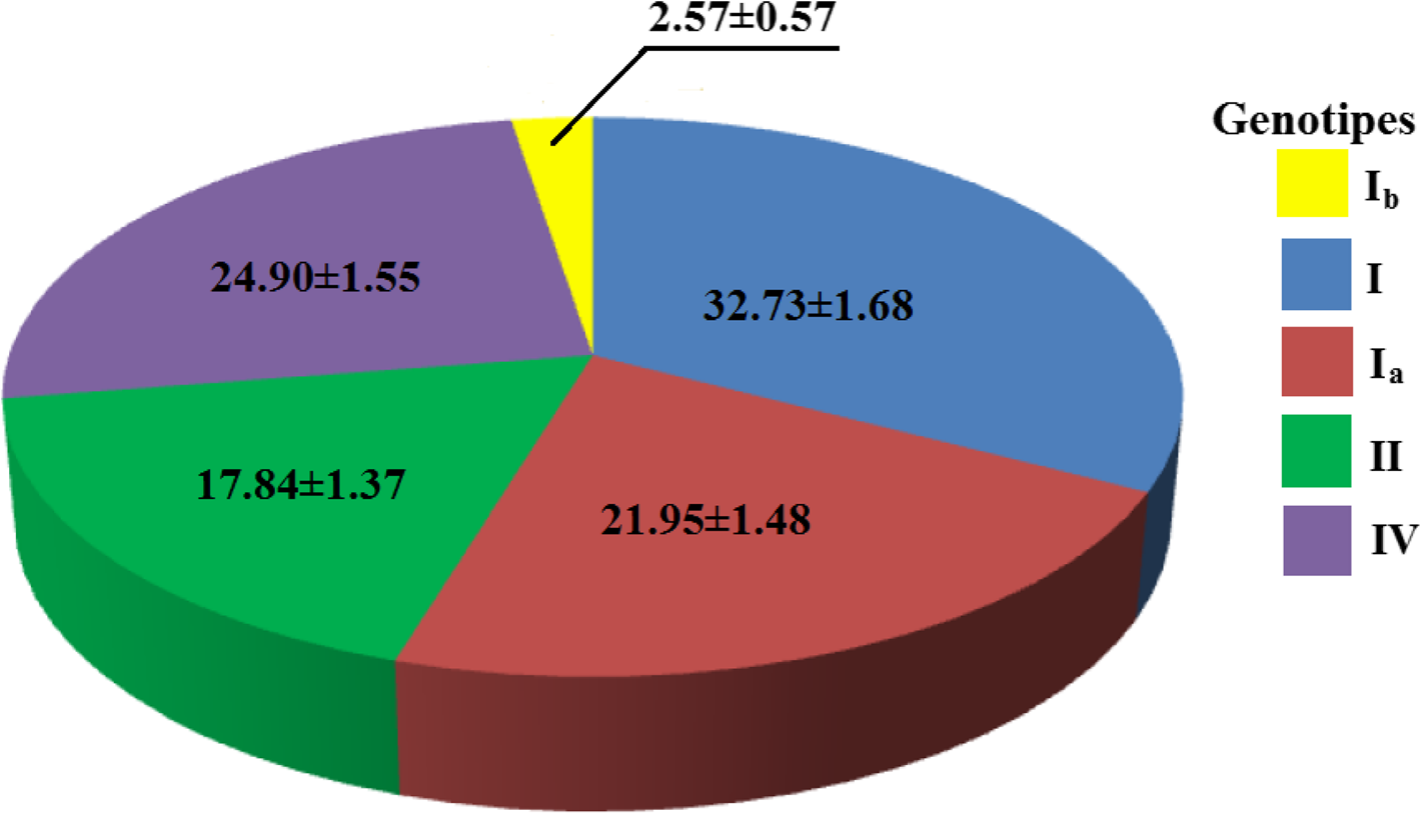
Frequency of *env* gene genotypes (%) in Western Siberia

The prevalence of the previously discovered and explored genotypes varied from 17.84 to 32.73% with the exception of the *I*_*b*_ genotype (Fig. 2). The number of samples with genotype *I*_*a*_ was 20.95 ± 1.46% (171 out of 779), which differed significantly (χ^2^ = 398; *P* < 0,001) from the statistical and methodological error and is equal to 1% [2]. The number of samples with genotype *I*_*b*_ was 2.57 ± 0.55% (20 of 779) and didn’t differ significantly from 1% (χ^2^ = 2.46). Therefore, of the two new genetic formations that were observed in the cattle of Siberia, only the *I*_*a*_ genotype is thought to occur normally (not mutant) in population frequency.

The highest number of leukocytes was observed in the blood of animals infected with the *BLV* of the genotype *I*. The hematological status was different in sick animals (P<0.01: P<0.001) and the total sample (P <0.001). The carriers of the *I*_*b*_ genotype were seen as the only exceptions; the difference in the number of leukocytes in the animals of the *I* genotype was not reliable (Table 1). The differences in the hematological status of the carriers of genotypes *I*_*a*_, *II,* and *IV* were not significant or they were of a low reliability (Р<0.05).

The carriers of the cattle leukemia virus *I*, *I*_*a*_, *II*, and *IV* genotypes were observed among animals that were sick, healthy, and those that were suspected of having leukemia. There was a heterogeneous relationship among the animals of different hematological statuses and animals infected by different virus genotypes (Table 2).

**Table 2:** Cytometric and morphological parameters of blood of animals-carriers of different genotypes of *BLV*

*env*	Hematological status	WBC, 10 ^9^/l				lymf, 10 ^9^/l				*n*
		$\overline{X}$	${S}_{\overline{x}}$	-5%	+ 5%	$\overline{X}$	${S}_{\overline{x}}$	-5%	+ 5%	
I	suspected	12.30	0.132	12.04	12.56	5.60	0.104	5.39	5.81	210
	sick	24.18	0.452	23.29	25.07	13.65	0.357	12.94	14.35	18
	healthy	4.95	0.369	4.22	5.68	2.45	0.292	1.88	3.03	27
	On average	12.36	0.180	12.01	12.71	5.83	0.140	5.55	6.12	255
I_a_	suspected	11.26	0.256	10.76	11.76	5.11	0.202	4.72	5.51	56
	sick	19.82	0.429	18.98	20.66	12.38	0.339	11.71	13.04	20
	healthy	7.06	0.197	6.67	7.44	3.08	0.155	2.77	3.39	95
	On average	9.93	0.240	9.44	10.40	4.83	0.190	4.45	5.21	171
II	suspected	9.73	0.195	9.35	10.11	4.09	0.154	3.79	4.40	97
	sick	17.40	1.109	15.22	19.57	9.36	0.876	7.64	11.08	3
	healthy	6.81	0.307	6.21	7.42	3.04	0.243	2.57	3.52	39
	On average	9.08	0.246	8.60	9.56	3.91	0.195	3.53	4.30	139
IV	suspected	11.70	0.233	11.24	12.16	5.44	0.184	5.08	5.80	68
	sick	17.25	0.960	15.36	19.13	10.50	0.759	9.00	11.99	4
	healthy	8.36	0.173	8.02	8.70	3.57	0.137	3.30	3.84	122
	On average	9.71	0.210	9.30	10.13	4.37	0.170	4.04	4.70	194
I_b_	suspected	11.99	0.429	11.14	12.83	5.68	0.339	5.018	6.35	20

Wilks’ lambda statistic = 0,82,285, F(12, 1530) = 13,056, *p*<0,001

WBC – number of white blood cells; lymf – number of lymphocytes $\overline{X}$ – the arithmetic average; ${S}_{\overline{x}}$ – error of the arithmetic average; −5 and + 5% are denoted the corresponding deviation of the attribute in the greater and lesser direction relative to the arithmetic mean; *n –* number of animals

The highest number of lymphocytes was observed in the blood of genotype *I* carriers, while the lowest number of lymphocytes was found in the animals with genotype *II* (Table 2). The similarity of the qualitative blood parameters of the cattle infected with *BLV* in different genotypes of the *env* gene, expressed through Wilks’ lambda statistic (0.82285), can be considered with some to be sufficiently high, but not identical.

**Fig. 3:**
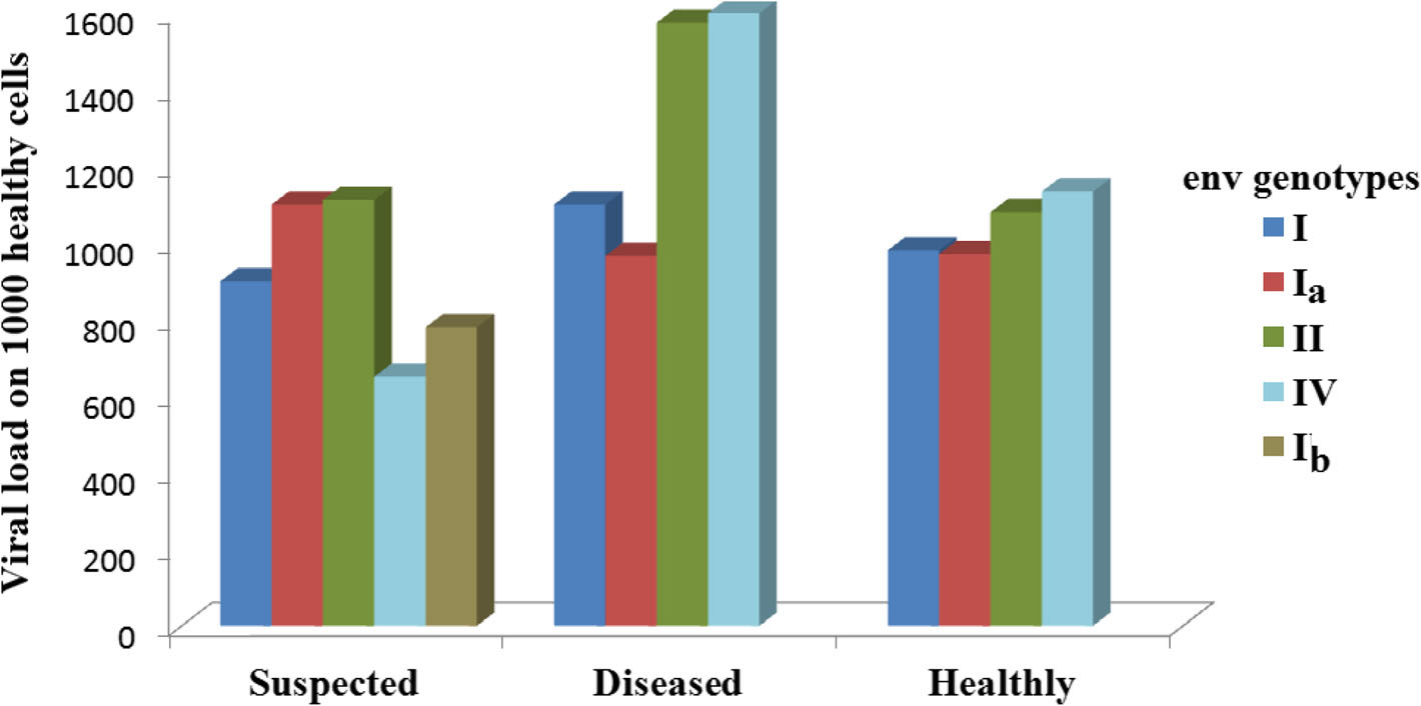
Distribution if viral load at different stages of infection progress in relation to heterogeneity of *env* gene *BLV* (휆Wilks’ =0,66,912, F = 28,406, *p* < 0.001; confidence intervals-95%)

The investigation into the bovine viral status had unexpected result. The typical sequence of viral load distribution for genotypes on the LRT-area [27] (sick > suspected > healthy) is not clearly shown in the present experiment (Fig. 3). Observed a non-typical relationship between the physiological status and viral status in the animals infected with genotype *I*. The maximum number of viral particles was observed in the blood of healthy animals, slightly lower in suspected animals, and minimally in animals with hematolytic leukemia (Fig. 3).

## Discussion

The current research does not prove that the higher the viral load, the higher the immune response of the leucocytes, despite the earlier results that showed that the genotypes of the LRT area affect the type of cattle leukosis [27]. The research shows that the highest number of leukocytes was observed in the blood of the cattle infected with the virus of *I* genotype (Table 1); the highest viral load was observed in the animals infected with *BLV* of *II*-and *IV* genotypes (Fig. 3.).

The research results are unique due to the high diversity of the discovered virus genotypes (4 genotypes of the standard frequency and 1 genotype of the frequency indistinguishable from 1%) (Table 2, Fig. 2). Early research detected one or two *BLV* genotypes of the *env* gene inone population of cattle [24, 28]. The observed diversity of the virus may be caused by the massive import of cattle to Siberia from other regions, particularly from abroad.

Interestingly, genotypes *I* and *IV* and their subtypes were found in *BLV* isolated from the biological material of cattle that were bred in different countries. Genotype *I* was found in the cattle from Japan, the United States, Australia, Germany, Korea, Iran, Brazil, Colombia, and the Dominican Republic [24, 29–34]. Genotype *IV* was observed in the cattle from Brazil, Belgium, Poland, Ukraine, and France. In Russia, and particularly in Siberia, only genotype *IV BLV* by the *env* gene was observed early [29, 33]. The cattle and sperm from Europe, the US, and Canada were delivered to Siberiain order to improve local livestock productivity [35, 36], the presence of the *BLV* genotypes *I* and *IV* in the explored sample is logically explained.

Interestingly the presence of the genotype *II*, peculiar only to South American cattle populations, had an frequency of 17.84% in the blood samples (Fig. 2). Cattle delivery from South American countries, where genotype *II* is widespread, to Siberia is not observed in the scientific literature. The cattle from South America have similar dairy productions to Russian cattle [37, 38]. The period of delivery and the different climate conditions between Siberia and South America assume lack of breeding and economic efficiency of black and white cattle when considering their delivery from South America to Siberia. Therefore, the introduction of genotype *II BLV* seems highly doubtful.

Phylogenetically, genotype *II BLV* is a separate complex that is distant from genotypes *I* and *IV* [39]. The research on isolated laboratory strains of *E. coli* [40] and red salmon inhabiting the rivers and lakes of Alaska [41] showed the independent directions of mutagenesis in the isolated populations. The model of allopatric speciation is based on geographical isolations [42] that helps divergence of morphogenetic characteristics of the strains, breeds, or populations [3, 43, 44]. Therefore, the possibility of an independent formation of the Siberian and South American *BLV* genotype *II* by convergent processes accepted as unlikely. It may be that genotype *II BLV* originated in a certain, presumably European or North American, cattle population and was then further introduced in the another regions. It is in Europe and North America that are the main suppliers of bull semen with a maternal productivity of 15,000 kg of milk per 305 days of lactation to other regions of the world [37]. Therefore, the assumption of the origin of the *II* genotype in the European or North American cattle population looks quite reasonable. Subsequently, probably, there was its independent introduction to Siberia and to South America together with importation of animals or a semen of bulls. However, this hypothesis, despite its logic, remains only a working version that requires additional experimental verification.

**Fig. 4:**
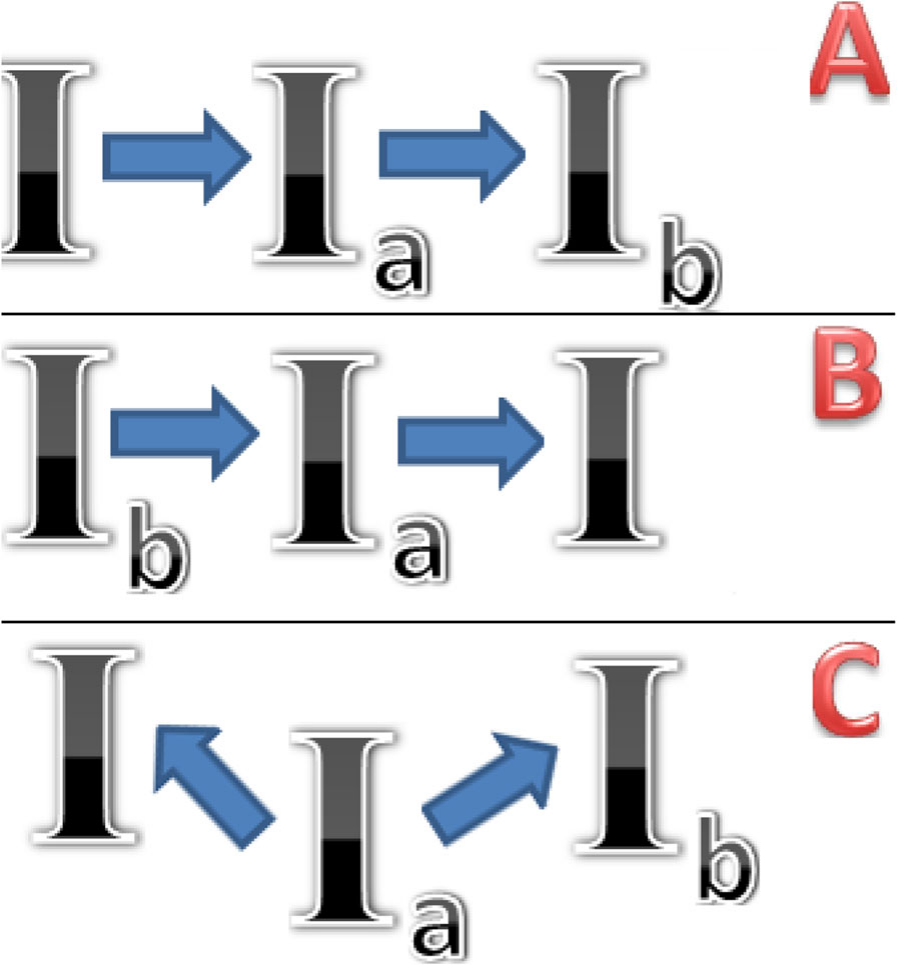
Hyphothetical schemes origination of *BVL* genotype I on *env* gene

The map of restriction fragments (Fig. 1) defines the line of mutations caused by genotype *I* to genotype *I*_*b*_ (Fig. 4a). This statement is arguable based on the controversial idea that if something had not been discovered before, then it did not exist. Our previous article and other papers [27, 45, 46] have highlighted the evolutionary advantage of more viral strains over less viral ones. Changes, as a rule, mostly aim at improving the functional properties of an object and its complex structural organization [3, 47]. Therefore, the general evolutionary vector should aim at increasing viral capacity it is supported by the rapid synthesis of viral particles and contributes to the evolutionary advantage of the strain as an intra specific generation. Analyzing the maximum number of leukocytes and the viral load, the observed genotype *I*_*b*_ as the least virulent and prevalent, followed by genotype *I*_*a*_. The virus with genotype *I* (Table 2, Fig. 3) caused the highest immune response.

Thus, the second hypothesis can be put forward for the chronology of *BLV* genotypes originating from the *env* gene, where genotype *I*_*b*_ can be considered as the initial form, and then, due to the accumulation of mutations, the strain *I*_*a*_ emerged, and the last and most progressive form is the carrier particles of genotype *I* (Fig. 4b).

Considering the analysis of restriction fragments (Fig. 1), there is third hypothesis which states that genotype *I*_*a*_ is the ancestral one and the mutations that formed genotypes *I* and *I*_*b*_ were accumulated independently (Fig. 4c). This model seems to be the least attractive as it admits the appearance and consolidation of genotype *I*_*b*_ with a frequency indistinguishable from the mutant one, with the lower number of viral particles than that of genotype *I*_*a*,_ and lower rate of their synthesis. This means a lower reproduction of a virus’sown copies and an evolutionary unattractiveness to this new formation.

Two of the three mentioned hypotheses make the case that genotypes *I*_*a*_ and *I*_*b*_ are not new growths that emerged in Western Siberia as a result of mutagenesis. They are relict forms of *BLV*, preserved in the cattle and displaced in other places by progressive strains.

## Conclusions

The research discovered of5 *BLV* genotypes in the *env* gene, where genotype *I*_*b*_ was observed with a frequency (2.57 ± 0.57%) indistinguishable from that of the mutant genotype (1%). Genotypes *I, I*_*a*_*, II, IV* were found to have a frequency of 17.84 to 32.73%. The highest number of leukocytes was observed in the blood of animals infected with the *BLV I* genotype. Hematological status differed in respect to sick animals (P<0,001) and the sample as a whole (P<0,001). The maximum viral load was observed in the carriers of genotypes *II* and *IV* (1000–1400 viral particles per 1000 healthy cells). The number of viral particles in the family *I* genotypes vary from 700 to 900. For the first time, the paper describes genotypes *I*_*a*_ and *I*_*b*_. Three hypotheses were suggested that try to explain the stages in the origination of genotype *I*. According to one of the hypotheses, genotypes *I*_*a*_ and *I*_*b*_ are new growths that originated in Western Siberia due to mutagenesis. Two other theories suggest that the explored genotypes are relict forms of *BLV* that have been displaced in the cattle by more progressive strains.

## Methods

Samples of whole blood were taken from black and -white Holstein cows (*n* = 779) located in the Novosibirsk region in Russia. The blood samples were obtained in May 2019 from the sub clavian vein using sterile catheters with EDTA added as an anticoagulant. Cytofluorometric and morphological parameters of the blood were determined by means of the RSE-90 Vet automatic veterinary hematological analyzer.

The total DNA was isolated by DNA-Sorb-B (Central Research Institute of Epidemiology, Russia) in order to conduct PCR analysis. The screening tests for observation of *BLV* in the blood samples were conducted by AmpliSens® (Central Research Institute of Epidemiology, Russia). The authors explored the *env* gene sequence by means of PCR-RPF analysis (*gp*51 fragment of *env* gene) according to the practice of the National Veterinary Institute (Poland, Pulava). Amplification was accomplished using the nest method in two stages with the use of 3 primers. The number of cycles, their temperature, and time parameters were set in agreement with the methodology of the National Veterinary Institute (Table 3). As the control strain was used FLK isolate The FLK strain (GenBank inventory number EF600696)*.*

**Table 3:** Temperature profile of PCR

Number of cycles	Temperature, °C	Period of time
1	95	3 min
34	95	30 s
	62	30 s
	72	2 min
1	72	10 min
Storage	4	< 12 h

The calculation of the number of components necessary for a reaction in line with the recommended number of samples was obtained according to the formula (1):

$$ M=m\times n+3+1 $$

Where, *m* is the amount of reaction mixture per a sample, *n* is the number of samples for virus tests, 3 is the number of controls in the reaction (IC = internal control for PCR, NC = negative control, PC = positive control), 1 is the safety amount of reaction mixture equal to the reaction mixture per a sample. FLK *BLV* was applied as the positive control and a DNA buffer was used as the negative control. Table 4 illustrates the sufficient number of components for each reaction.

**Table 4:** Composition of reaction mixture

Mixture components	Necessary amount, mcl	
	Reaction1	Reaction2
10хof optimized DNA-buffer	5.0	5.0
10 mM MgCl_2_	1.0	1.0
10 mM dNTP	1.0	1.0
10 mM primerAP (direct)	1.0	1.0
10 mM primerZM2 (reverse)	1.0	1.0
2 U/μLTAGpol	1.0	1.0
Water, ml	13.0	10.0
DNA, 50 ng	2.0	2.0

Primers from Table 5 were produced by an automated synthesizer of oligonucleotides that was purchased from the “SibEnzyme” (Novosibirsk, Russia). The accuracy of the рrimers was defined by the HPLC method and its accuracy was at least 95%.

**Table 5:** Primers used for PCR analysis

Title of the primer	Sequence of oligonucleotides (5′- > 3′)	Site of flanking beginning	Site of flanking end
Forvard primer AP	GCTCTCCTGGCTACTGACC	4772	791
Reverse primer ZM2	CTCTGATGGCTAAGGGCAGACACGGC	5822	848
Reverse primer ZM5	GCTAGGCCTAAGGTCAGGGCCGC	5743	766

The products of amplification were analyzed by means of fragment restriction acceleration that was caused by the horizontal electrophoresis method in agarose gel. Samples suspected of short fragments were additionally detected by vertical electrophoresis in polyamide acrylic gel [48]. The restriction was realized by the enzymes *HaeIII* and *BstYI*, which were produced by the “SibEnzyme”. Restriction fragments, which were used to determine the genotype of the virus (Table 1), were compared with fixed-length nucleotide sequences, which were synthesized to order in the company “SibEnzyme”.

The significance of the differences was determined by the Student’s criterion [49]. The error of genotype frequencies was calculated by Zhivotovsky’s formula [50]. Comparison of genotype frequencies with the maximum permissible mutant allele frequency of 1% [2] was carried out using the Chi-square criterion [49]. Discriminant analysis was conducted using STATISTICA 10 software.

## Data Availability

All data generated or analyzed during this study are included in this published article.

## Abbreviations

*BLV*: Bovine Leukemia Virus
PCR-RFLP: Polymerase Chain Reaction-Restriction Fragment Length Polymorphism
IC: Internal control
NC: Negative control
PC: Positive control
LRT-area: Long terminal repeats –the area of the *BLV* genome
gp: glycoprotein
bp: Bases pair
